# Quantitative Single-Cell Transcript Assessment of Biomarkers Supports Cellular Heterogeneity in the Bovine IVD

**DOI:** 10.3390/vetsci6020042

**Published:** 2019-05-12

**Authors:** Kangning Li, Devin Kapper, Sumona Mondal, Thomas Lufkin, Petra Kraus

**Affiliations:** 1 Department of Biology, Clarkson University, 8 Clarkson Ave, Potsdam, NY 13699, USA; kali@clarkson.edu (K.L.); tlufkin@clarkson.edu (T.L.); 2 Department of Mathematics, Clarkson University, 8 Clarkson Ave, Potsdam, NY 13699, USA; kapperdp@clarkson.edu (D.K.); smondal@clarkson.edu (S.M.)

**Keywords:** intervertebral disc, annulus fibrosus, nucleus pulposus, gene expression, quantitative, heterogeneity, RNA in situ hybridization, fluorescence, biomarker

## Abstract

Severe and chronic low back pain is often associated with intervertebral disc (IVD) degeneration. While imposing a considerable socio-economic burden worldwide, IVD degeneration is also severely impacting on the quality of life of affected individuals. Cell-based regenerative medicine approaches have moved into clinical trials, yet IVD cell identities in the mature disc remain to be fully elucidated and tissue heterogeneity exists, requiring a better characterization of IVD cells. The bovine coccygeal IVD is an accepted research model to study IVD mechano-biology and disc homeostasis. Recently, we identified novel IVD biomarkers in the outer annulus fibrosus (AF) and nucleus pulposus (NP) of the mature bovine coccygeal IVD through RNA in situ hybridization (AP-RISH) and z-proportion test. Here we follow up on *Lam1*, *Thy1*, *Gli1*, *Gli3*, *Noto*, *Ptprc*, *Scx*, *Sox2* and *Zscan10* with fluorescent RNA in situ hybridization (FL-RISH) and confocal microscopy. This permits sub-cellular transcript localization and the addition of quantitative single-cell derived values of mRNA expression levels to our previous analysis. Lastly, we used a Gaussian mixture modeling approach for the exploratory analysis of IVD cells. This work complements our earlier cell population proportion-based study, confirms the previously proposed biomarkers and indicates even further heterogeneity of cells in the outer AF and NP of a mature IVD.

## 1. Introduction

Cell-based tissue regeneration approaches to address degeneration of intervertebral discs (IVD) are a glimpse of hope for a large percentage of the population suffering from severe and chronic lower back pain due to age or injury related degenerative disc disease (DDD). The IVD is a tissue of strength, resilience and capable of reducing the impact of movement on the rigid vertebral bodies that stabilize our spine [1]. In this field of regenerative medicine, strategies are pursued to refurbish ailing IVDs with autologous or allogenic cells, derived either from the disc itself or other sources, with special interest in mesenchymal stem cells (MSC) derived from bone marrow, articular cartilage or adipose tissue. Alternatively, strategies to stimulate intrinsic disc cells through the injection of growth factors or the use of gene therapy to replace degenerating extracellular matrix (ECM) molecules are envisioned as ways to “rejuvenate” IVD function as a shock absorber in the spinal joints [2,3,4,5,6]. On first glance the IVD might appear as an anatomically simple structure, comprised of concentric rings in the annulus fibrosus (AF) and gradually transitioning towards a central nucleus pulposus (NP) [1,7,8]. However, compared to other vertebrate organs, the IVD is unique in many ways: The avascular nature of the IVD is reflected in a lack of coloration (see Figure 1A in [3]) and the few remaining cells in the mature IVD are embedded in a vast amount of ECM, likely experiencing only minor cell-cell contact [3]. These few resident cells within the outer AF and NP of mature IVDs display a heterogeneous profile when assessed on a single-cell level for their transcriptional activities [2,3]. Given several ongoing clinical trials [5,9,10,11] and an alarming emergence of stem cell tourism worldwide [12] there remains a need to further characterize these cells on a molecular level, should they be the subject or target of regenerative treatments [3,13,14,15]. 

Animals, especially *Mus musculus*, have long been extremely helpful as model organisms to study human development and genetic mutations underlying congenital diseases [16]. While important work has been accomplished in mouse to understand the early notochord to NP transition [17,18,19], the cell composition of the NP of mature caudal IVDs differs significantly between human and rodents or porcine [3,13,20] and points to alternative animal models like *Bos Taurus.* Respecting the 3R guidelines in research—replacement, reduction, and refinement—bovine tails are an ideal IVD source, as abattoirs often discard them. Bovine coccygeal discs provide a very suitable research model to study cell populations of the mature healthy IVD (Figure 1 in [20]). The coccygeal bovine IVD of a skeletally mature animal is considered similar to a human lumbar disc of a healthy young adult on an anatomical, histological, biochemical and biomechanical level [13,20,21,22,23,24] and represents an ethically more acceptable tissue source to study healthy cells compared to human IVD tissue. 

In need for further characterization of resident cells in the mature IVD, we recently proposed a set of novel IVD biomarkers based on the proportion of cells within the outer AF and NP tissue of bovine coccygeal IVDs being either positive or negative for the proposed biomarker transcript [3]: Laminin1 (Lam1) belongs to a group of glycoproteins of high molecular weight and is present in the ECM of the basal lamina with the ability to bind to collagens, integrins and proteoglycans [25]. Glioma-associated oncogene 1 (Gli1) and 3 (Gli3) belong to a family of transcription factors (TF) known as downstream mediators of hedgehog signaling [26,27,28]. Notochord (Noto) is a homeobox TF involved in early notochord development, acts downstream of brachyury [29] and is conserved during notochord development. Noto cell lineage tracing in mouse indicated that the NP originates from the notochord [30]. Scleraxis (Scx) is a basic helix-loop-helix TF otherwise found in connective tissues including tendons and ligaments and is implicated in skeletogenesis during mouse embryonic development [31,32]. Sex determining region Y-box 2 (Sox2) is essential for pluripotency of stem cells and involved with self-renewal capacity [33,34]. Zscan10 (Zinc finger and SCAN (*SRE-ZBP, CTfin51, AW-1* and Number 18 cDNA) domain containing) is a TF and proposed multipotency marker in mouse [35]. Tyrosine phosphate receptor type C (Ptprc or CD45) and thymocyte differentiation antigen 1 (Thy1 or CD90), are part of a marker panel defining multipotent mesenchymal stromal cells [36,37].

Analyzing these genes with RNA in situ hybridization (RISH), we point to heterogeneity among cells within the outer AF or NP, which is typically not accounted for by methods involving cell pooling for RNA extraction, such as qRT-PCR, microarray expression profiling or non-single-cell RNA sequencing [2,3,38]. Here, we also explore the use of fluorescent (FL) transcript tagging to allow for transcript quantification of proposed biomarkers through both population averaging and single-cell analysis and we propose that this analysis based on FL values enables further evaluation of cellular heterogeneity within the population of cells actively transcribing a biomarker. Lastly, we provide evidence that transcriptional heterogeneity in the mature IVD is not simply attributable to cells undergoing senescence.

## 2. Materials and Methods 

All procedures were performed according to ethical standards of Clarkson University (NIH Office of Laboratory Animal Welfare PHS Approved Animal Welfare Assurance Clarkson University-Assurance Number D16-00780 (A4536-01). No human material was included in this study. 

### 2.1. Tissue Collection and IVD Isolation

Tails of skeletally mature bovine animals were retrieved fresh from local abattoirs, transported on ice and processed within two hours. All procedures were carried out strictly under ribonuclease free conditions [39]. Coccygeal IVDs were isolated and fixed in 4% (w/v) paraformaldehyde (PFA), dehydrated through a gradient of ethanol baths and embedded in paraffin [40]. Sections with a thickness of 7 µm were cut on a rotary microtome and mounted on VistaVision^TM^Histobond^R^ glass slides (VWR, Radnor, PA, USA) [41].

### 2.2. Scanning Electron Microscopy (SEM)

IVDs were fixed overnight using 2.5% (v/v) glutaraldehyde (EMS, Hatfield, PA, USA) in 0.1 M sodium cacodylate solution (EMS) at 4 °C, followed by 0.1 M sodium cacodylate incubation at 4 °C overnight. Then the IVDs were gently dehydrated in 50%, 60%, 70%, 80%, 90%, and 100% ethanol baths. Initial air-drying was followed by freeze-drying overnight before the IVDs were Au/Pd sputter coated and examined with a JEOL JSM-7400F scanning electron microscope (JEOL Ltd., Akishima, Tokyo, Japan) at 1500× magnification.

### 2.3. Fluorescent RNA in situ Hybridization (FL-RISH) and Immunohistochemistry (IHC)

Templates for RNA probes were amplified by polymerase chain reaction (PCR) from bovine genomic DNA as described [3]. The enzymatic activity of T7 RNA polymerase facilitated labeling of RNA antisense probes with a digoxigenin (DIG) epitope (Roche, Basel, Switzerland) essentially as described in [20]. The epitope was detected by an anti-digoxin primary antibody (JIR, West Grove, PA, USA) and visualized with an Alexa-488 secondary antibody (JIR), while nuclei were stained with 4′,6-diamidino-2-phenylindole (DAPI) (Thermo Fisher, Waltham, MA, USA). Experiments omitting the use of a probe or the primary antibody served as controls for unspecific binding. For a combined FL-RISH/IHC analysis, a polyclonal rabbit anti-Ki67 antibody (Thermo Fisher) was added after the RISH washing steps and visualized with an Alexa-647 secondary antibody (Thermo Fisher). To visualize subcellular locations of mRNA transcripts as well as for Ki67 protein detection, cells were imaged at 63× magnification with zoom factor 3 using a DMi8 confocal microscope (Leica, Wetzlar, Germany).

### 2.4. Selection of Investigated Biomarkers

Previously we had investigated 50 genes based on their occurrence in IVD related literature [17,36,42,43,44] or based on data from our work in *Mus musculus* [9,35,45,46,47,48,49,50]. Through RNA in situ hybridization (AP-RISH) and z-proportion test we had identified a total of three predominantly outer AF and fifteen predominantly NP associated transcripts, confirming several existing biomarkers such as *Col1a1* in the outer AF and *Acan* in the NP. Of those 18 genes, two were novel biomarkers in the outer AF (*Lam1* and *Thy1*) and eight in NP tissue (*Gli1, Gli3*, *Noto*, *Scx*, *Ptprc*, *Sox2*, *Zscan10* and *Klhl14as* (*LOC101904175)*) [3]. Here, using FL-RISH in a modified approach, we extended our analysis of the nine protein-encoding novel biomarkers to provide quantification of mRNA transcript levels per cell.

### 2.5. Data Collection and Analysis

For transcript quantification of each proposed biomarker we chose 20 random fields of view within the outer AF and NP of three IVDs to capture fluorescent signal intensities, similar to the approach taken for *Col2a1* and *LOC101904175* [3]. Quantitative transcript data was collected under 40× magnification on a DMi8 confocal microscope (Leica) as maximum projection of four z-stack images. Background was accounted for through thresholding in Image J. Total fluorescence for each cell was calculated in Image J as the product of mean fluorescence and area values. For each biomarker and IVD, the mean total fluorescence was established for cells in the outer AF and NP, analyzed for significance using Student’s t-test and graphed with GraphPad PRISM 5 (GraphPad, San Diego, CA, USA). 

The Kernel density estimates of the data were graphed in R using a log2 transformation of the total fluorescence intensity for each transcriptionally active cell. The Shapiro–Wilk test was employed to assess the validity of a normality assumption for the data collected. When the data was not normally distributed, k-means clustering was performed to see if the data could be fit to a mixture of Gaussian populations using the expectation-maximization (EM) algorithm. Subsequently, if cells were found to be normally distributed within the sub-clusters, no further subdivision was pursued and the resulting Gaussian mixture model (GMM) was kept. Of note, we have excluded the possibility of artificial sub-clustering due to technical errors through natural data clustering. That is, we found those clusters to be uncorrelated with the frame number or individual IVD sections (Figure S1).

## 3. Results

### 3.1. Morphology of Outer AF and NP Cells in their ECM Environment

**Figure 1:**
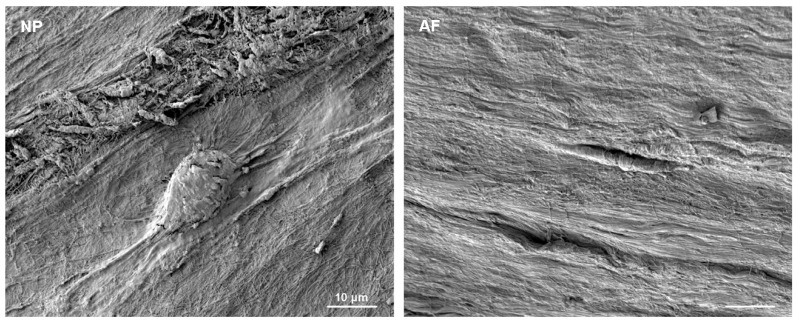
Scanning electron microscope (SEM) image of a mature bovine nucleus pulposus (NP) and outer annulus fibrosus (AF) indicates different cell morphologies and the vast extent of extracellular matrix (ECM) those cells reside in. Scale bar reflects 10 μm and is equivalent for both photos.

**Figure 2:**
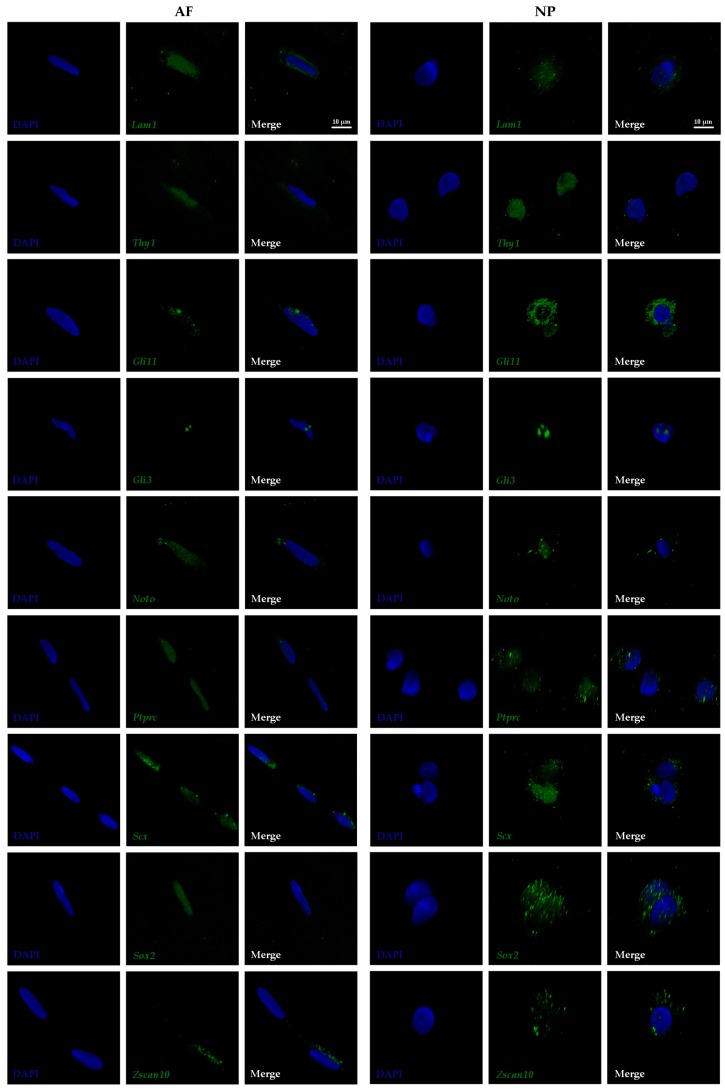
Fluorescent RNA in situ hybridization (FL-RISH) showing the transcripts (green) of nine proposed biomarkers with subcellular resolution in the outer AF and NP of a mature bovine intervertebral discs (IVD) at 63× magnification and zoom factor 3. Nuclei are visualized with DAPI (blue). Laminin1 (*Lam1*)*;* Thymocyte differentiation antigen 1 (*Thy1*); Glioma-associated oncogene 1 (*Gli1*); Glioma-associated oncogene 3 (*Gli3*); Notochord (*Noto*); Tyrosine phosphate receptor type C (*Ptprc*); Scleraxis (*Scx*); Sex determining region Y-box 2 (*Sox2*); Zinc finger and SCAN (*SRE-ZBP, CTfin51, AW-*1 and Number 18 cDNA) domain containing transcription factor 10 (*Zscan10*). Scale bar reflects 10 μm.

Cells of the mature AF and NP reside in a large amount of ECM. Fibers of structural molecules in the ECM appear aligned in a linear manner in the outer AF and show a more crisscross pattern in the NP (Figure 1). NP cells are typically of round appearance and often described as chondrocyte-like. Larger and smaller cell bodies are seen side-by-side indicating morphological heterogeneity. Often long, bulging cell protrusions are visible in vivo and in vitro (Figure 1 and [2,20]). Cells in the AF are elongated, resembling more the shape of fibroblasts (Figure 1). This difference in cell morphology was also reflected by the shape of the cell nuclei, which is typically elongated in cells of the outer AF and round in NP cells as seen after DAPI staining (Figure 2). 

### 3.2. Subcellular Transcript Localization of Proposed Biomarkers in Outer AF and NP Cells

Owing to the high resolution of confocal imaging and the availability of 3D manipulation software tools, the intracellular location of gene transcripts could be visualized after FL-RISH and imaging at 63× magnification (Figure 2). According to the dogma of biology, primary protein encoding mRNA transcripts are first generated in the nucleus, processed and then exported into the cytoplasm for translation. As shown here, some transcripts were mostly localized in the nucleus (*Thy1*, *Ptprc* and *Sox2* in cells of the outer AF), some were found predominantly in the cytoplasm (*Gli1* in outer AF cells and *Zscan10* in both cell types analyzed) and others were typically found in both cellular compartments (*Thy 1* in outer AF cells, *Gli1, Ptprc* and *Sox2 in NP cells* or *Gli3, Noto* and *Scx* in both cell types) (Figure 2). The intracellular transcript localization of the transcription factors *Gli1*, *Gli3*, *Sox2* and *Zscan10* showed distinct bright foci, while transcripts of the other genes analyzed often had a more homogeneous appearance (Figure 2).

### 3.3. Confirmation of Biomarkers Using Transcript Quantification Through Population Averaging

**Figure 3:**
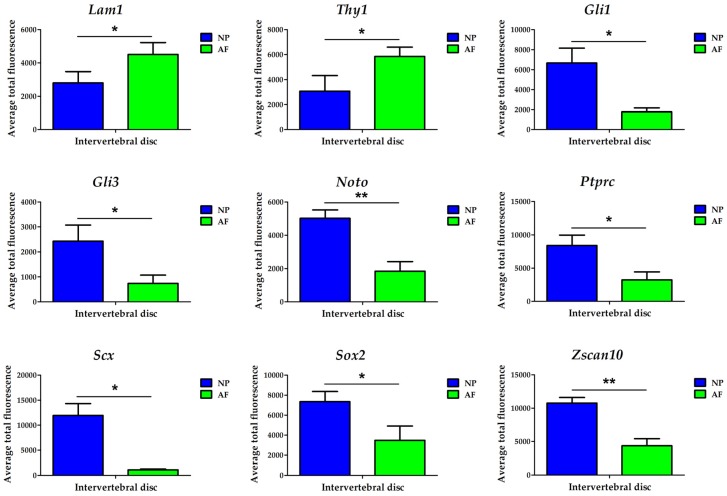
Quantification of nine proposed biomarkers in the outer AF (green) and NP (blue) of a mature bovine IVD using a population averaging approach. Statistical significance was determined using Student’s t-test. Cell types are reflected on the x-axis while the average total fluorescence is displayed on the y-axis. * *p* < 0.05 and ** *p* < 0.01. Laminin1 (*Lam1*); Thymocyte differentiation antigen 1 (*Thy1*); Glioma-associated oncogene 1 (*Gli1*); Glioma-associated oncogene 3 (*Gli3*); Notochord (*Noto*); Tyrosine phosphate receptor type C (*Ptprc*); Scleraxis (*Scx*); Sex determining region Y-box 2 (*Sox2*); Zinc finger and SCAN (*SRE-ZBP, CTfin51, AW-1* and Number 18 cDNA) domain containing transcription factor 10 (*Zscan10*).

We further investigated our recently proposed protein-encoding biomarkers in the outer AF (*Lam1* and *Thy1*) and NP tissue (*Gli1, Gli3, Noto, Scx, Ptprc, Sox2* and *Zscan10*) using fluorescent tagging of the transcripts (FL-RISH) and confocal imaging (Figure 3 and Table 1 and Table S1). We first used the approach of identifying positive and negative cells and subjected these data to z-proportion analysis (Table 1). Next, using transcript quantification per cell, and comparing the mean of the cell population average for total fluorescence in the outer AF and NP, the proposed outer AF biomarkers *Lam1* and *Thy1* alongside the proposed NP biomarkers were confirmed as significant using data generated by FL-RISH (Figure 3, Table 1 and Table S1).

### 3.4. Transcriptional Heterogeneity in IVD Cells is Not a Sole Consequence of Cell Cycle Arrest

**Figure 4:**
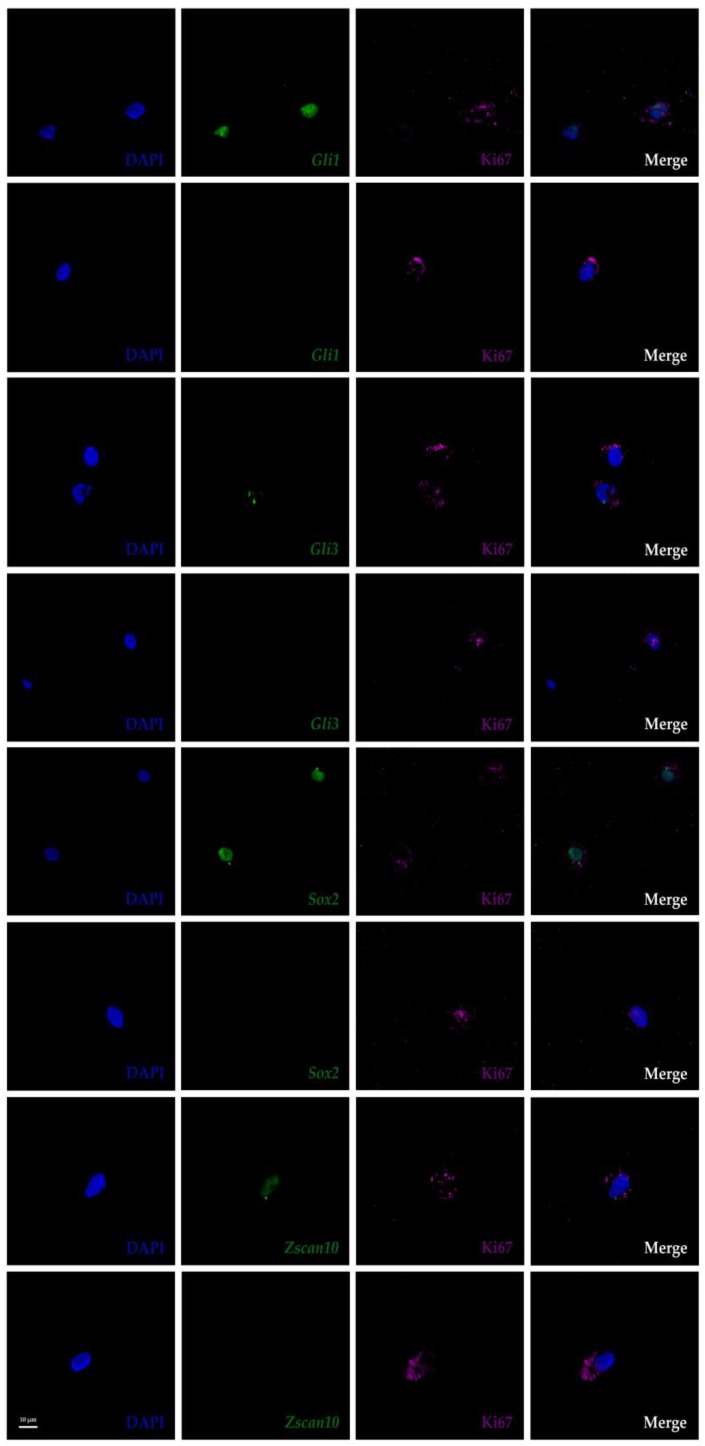
Combined FL-RISH and immunohistochemistry (IHC) showing RNA transcripts (green) and Ki67 protein (magenta) in cells of the nucleus pulposus of a mature bovine IVD. DAPI (blue) labels the nucleus. Ki67 indicates active phases of the cell cycle, or cells that are not in G0). Images shown at 63× magnification and zoom factor of three. Glioma-associated oncogene 1 *(Gli1)*; Glioma-associated oncogene 3 *(Gli3)*; Sex determining region Y-box 2 *(Sox2*); Zinc finger and SCAN (*SRE-ZBP, CTfin51, AW-1* and Number 18 cDNA) domain containing transcription factor 10 (Zscan10). Scale bar reflects 10 μm.

Heterogeneity was confirmed with the FL-RISH approach as shown for *Zscan10* expression in the AF and *Ptprc* expression in the NP (Figure 2) and by z-proportion analysis (Table 1). We also performed a combined FL-RISH and FL-IHC analysis using Ki67 as an indicator for cells in the active phase of the cell cycle (Figure 4). As shown here for *Gli1*, *Gli3*, *Sox2* and *Zscan10,* we found Ki67 positive NP-cells in both the presence and absence of these transcripts. 

### 3.5. Single Cell Transcript Analysis Indicates Further Heterogeneity in Transcriptionally Active Cells.

**Figure 5:**
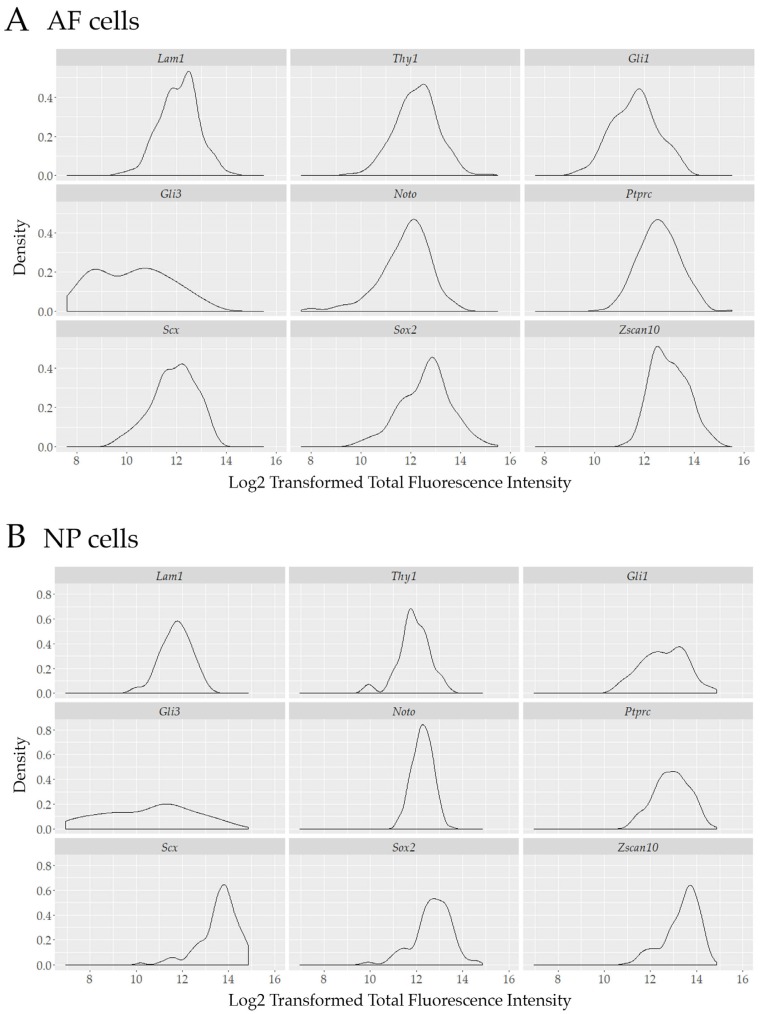
Kernel density estimates for nine proposed biomarkers in (**A**) cells of the outer AF and (**B**) NP of a mature bovine IVD illustrating the potential heterogeneity within the group of transcriptionally active cells. Cell density on the y-axis is plotted against the log2 transformation of the total fluorescence intensity on the x-axis. Laminin1 (*Lam1*); Thymocyte differentiation antigen 1 (*Thy1*); Glioma-associated oncogene 1 (*Gli1*); Glioma-associated oncogene 3 (*Gli3*); Notochord (*Noto*); Tyrosine phosphate receptor type C (*Ptprc*); Scleraxis (*Scx*); Sex determining region Y-box 2 (*Sox2*); Zinc finger and SCAN (*SRE-ZBP, CTfin51, AW-1* and Number 18 cDNA) domain containing transcription factor 10 (*Zscan10*).

**Figure 6:**
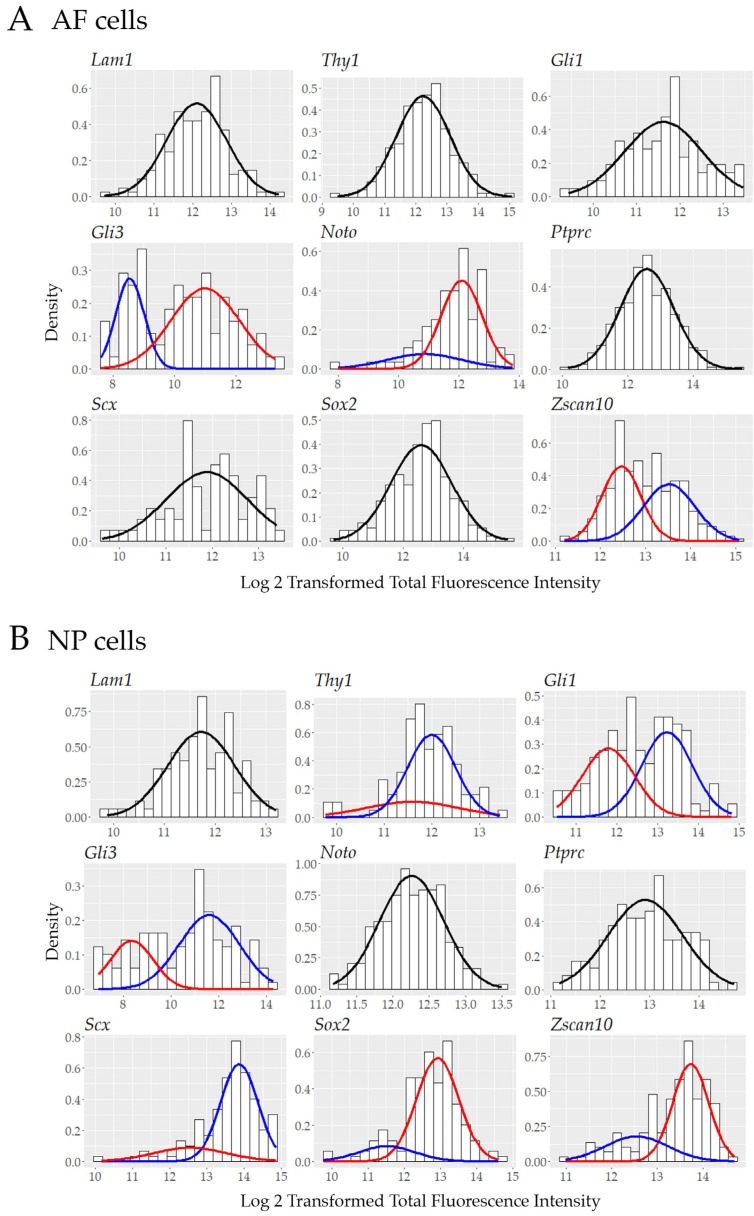
Gaussian mixture model and histograms of data for nine proposed biomarkers in (**A**) cells of the outer AF and (**B**) NP of a mature bovine IVD fitted to one or two Gaussian distributions using the expectation-maximization algorithm depending on the normality of the data. Laminin1 (*Lam1*); Thymocyte differentiation antigen 1 (*Thy1*); Glioma-associated oncogene 1 (*Gli1*); Glioma-associated oncogene 3 (*Gli3*); Notochord (*Noto*); Tyrosine phosphate receptor type C (*Ptprc*); Scleraxis (*Scx*); Sex determining region Y-box 2 (*Sox2*); Zinc finger and SCAN (*SRE-ZBP, CTfin51, AW-1* and Number 18 cDNA) domain containing transcription factor 10 (*Zscan10*)

To further investigate the pool of transcriptionally active cells and to provide assessment of cell heterogeneity with single-cell resolution, the total fluorescence intensity of each positive cell was graphed using a Kernel density distribution plot. Some of our biomarkers showed a non-normal distribution in transcriptionally active outer AF cells: *Gli3*, *Noto*, and *Zscan10* (Figure 5A) or NP cells: *Thy1*, *Gli1*, *Gli3*, *Scx*, *Sox2* and *Zscan10* (Figure 5B) as determined through the use of the Shapiro–Wilk test; k-means clustering was performed when data was not normally distributed to fit data to a Gaussian mixture model (GMM) (Tables S2 and S3). Using this approach and assessing one gene at a time we identified distinct sub-populations with at least a two-fold difference of transcript levels as represented by the total fluorescence intensity of *Gli3, Noto* and *Zscan10* expressing cells in the outer AF cell population (Figure 6A) and for *Thy1, Gli1, Gli3, Scx, Sox2* and *Zscan10* in the NP cell population (Figure 6B).

## 4. Discussion

Discogenic, severe and chronic lower back pain due to disc trauma and degeneration is a debilitating condition that patients, once affected, often have to endure for a significant part of their life. So far, partial alleviation of symptoms but no complete cure or full restoration of athletic abilities or lifestyle choices is available [51,52]. Cell therapy-based regenerative medicine holds promises to delay or revert the degeneration of aging or injured body parts and animal studies as well as clinical trials for IVD regeneration are ongoing (www.clinicaltrials.gov) [9,10,11,53]. At the same time a more profound understanding of the pathophysiology of DDD and the cell types involved is required [37]. DDD is accompanied by a decrease in proteoglycan and collagen II synthesis alongside the degeneration of existing collagen II and an increase of collagen I in the NP [15,54,55,56,57,58]. In the context of pathophysiology, a recent study suggested a link between iron deficiency and DDD [59], while on the contrary, an iron-overload and related free radical generation was considered a factor leading to DDD in patients with beta thalassemia major [60]. With regards to IVD development, progress has come from transgenic mouse models pointing to the contribution of notochord cells to the NP; however, in the mature human or bovine NP those cells are not retained to the same extent as they are in mouse or other rodents [3,17,18,19,20,61,62]. The degeneration of ECM macromolecules like glycosaminoglycans (GAG) and the resulting loss of hydration could be a consequence thereof [7,8,11,55,63,64]. Nowadays human NP and AF cells are commercially available (www.sciencellonline.com), however their original fate and gene expression patterns might change in vitro without appropriate ECM and 3D-culture systems in place, as seen for primary bovine cells derived from IVD tissue and maintained in monolayer culture [20]. Furthermore, ethical concerns preclude transgenic lineage tracing of human IVD cells or higher primates in vivo, and not all animal models studied provide accurate cell composition, disc size or loadbearing conditions in the adult [11,52]. The bovine coccygeal IVD is an accepted research model to study cells in the environment of a healthy IVD, resembling closely the histological characteristics of a human lumbar IVD from a healthy young adult [20,21,22,23]. 

Aside from their exposure to mechanical, physical and chemical constraints [55,65,66,67,68] AF and NP cells exist like eremites in unique microenvironments. Cells in the mature IVD are sparse, either isolated or present as small cell clusters embedded in a vast amount of ECM (Figure 1) and [3,58,69], with their survival depending on the diffusion of nutrients or signaling cues through the extensive meshwork of ECM molecules [13,70,71]. Morphological heterogeneity has been described: NP cells are typically of round appearance with larger and vacuoled or smaller cell bodies, notochord cell-derived or chondrocyte-like, while cells in the AF are elongated, resembling more the shape of fibroblasts (Figure 1) and [3,6,61,62,69,72,73,74]. An ongoing debate concerns the fate of notochord cells during maturation of the human or bovine NP [13,62,75]. Are those cells replaced over time by migrants from adjacent AF or endplate tissue, as suggested in a mouse injury model [76], and do they subsequently undergo a change in morphology in response to differences in ECM stiffness and fiber alignment between the AF and NP, or do notochord cells eventually differentiate into small chondrocyte-like cells as indicated by qRT-PCR on two morphologically different cell populations which maintain expression of genes like *KRT19* and *T* (brachyury) as a population [13,37]. We have previously identified positive and negative cells for *T* expression in cells of the AF and NP, however, with no significant difference in cell population proportions, indicating that assessment of heterogeneity within a cell population remains important to address the question of NP cell origin and heterogeneity [3], whether within their native environment or in culture. We have frequently isolated and propagated bovine cells from the outer AF and the NP through non-enzymatic explant outgrowth culture [20,77]. These cells exhibit some criteria established for mesenchymal stem cells, but continue to display heterogeneity when phenotyped by a selection of transcripts, by morphology or by cell movement [2,20,77,78,79]. In this context others and we find morphological, molecular and “behavioral” differences in vivo and in vitro pointing to at least two cell populations within the outer AF and NP [2,3,61,68,73]. Since a more complex heterogeneity is apparent on the transcriptome level, the molecular identities and developmental origins of cells remaining in the healthy mature human or bovine IVD still need to be addressed further. Hence, the definition of biomarkers to identify AF and NP cells is of strong interest to the field [3,20,44,61,80,81]. In an attempt to contribute to the molecular characterization of IVD cells, we recently proposed a set of novel bovine IVD biomarkers out of 50 genes investigated by chromogenic AP-RISH using a cell population proportion based approach, where transcriptional heterogeneity was indicated through cells expressing (denoted positive) or not expressing (denoted negative) a gene transcript within the same tissue type [3]. These genes were expressed in a significantly larger proportion of cells in the outer AF (*Lam1*, *Thy1*) or NP (*Gli1*, *Gli3*, *Noto*, *Ptprc*, *Scx*, *Sox2* and *Zscan10)*. Other biomarkers previously proposed by others in the AF, like *Col1a1* or in the NP, like *Acan*, *Col1a2*, *Col2a1*, *Krt18*, *Krt19*, *Shh* and *Ca12* were also confirmed with our approach, while some did not show any significant difference in the proportion of positive cells between the outer AF and NP, for example *Foxf1*, *Hif1a*, *Krt8, Snap25, Sox5, Sox6, Sox9, Pax1*, *T* and *Tmnd*. Since our approach to analyze cell population proportions had differed from the common practice of cell pooling and expression profiling of cell population averages which uses methods like microarray, RNAseq or qRT-PCR [42,43,44],we developed here a modification of AP-RISH by using fluorescently tagged RNA probes in combination with statistical data analysis that facilitated both, analysis by cell averaging to be comparable to existing data and single-cell assessment. For comparison of our AP- and FL-RISH data, we initially also identified positive and negative cells and subjected the data to z-proportion analysis and observed a similar trend as described before (Table 1 and [3]). The FL-RISH based method can be applied in vitro and to cells in their natural surroundings in vivo, as demonstrated here. Both RISH methods confirmed *Lam1* and *Thy1* as potential AF and *Gli1, Gli3, Noto, Scx, Ptprc, Sox2 and Zscan10* as potential NP biomarkers. To demonstrate that the observed transcriptional heterogeneity cannot simply be attributed to cells leaving the active phase of the cell cycle, we used the widely accepted cell proliferation marker Ki67 [82,83] in combination with FL-RISH analysis of our proposed biomarkers. We have demonstrated that cells without mRNA expression of the analyzed biomarker are not necessarily in G0. Instead, this transcriptional heterogeneity supports sub-populations within outer AF and NP cells. Additionally, by using a combination of FL-RISH and a GMM approach to further analyze the transcriptionally active cells in their native environment, we noticed for some transcripts in cells of the NP (*Thy1*, *Gli1*, *Gli3*, *Scx*, *Sox2* and *Zscan10)* or the outer AF (*Gli3, Noto* and *Zscan10),* additional heterogeneity based on fluorescent intensities and hence transcript levels. While neither *Gli3*, *Noto,* nor *Zscan10* are considered AF-specific, neither is *Thy1* considered a NP biomarker, the indication of further heterogeneity could point to mixed cell sub-populations of shared developmental origin.

Despite strong evidence pointing to heterogeneity of outer AF and NP cells in the mature IVD, currently most expression profiling data available for the IVD is based on cell pooling and population averaging technologies [14,42,43,44,81,84]. Here, by using FL-RISH with single cell resolution, we focused on the further evaluation and quantification of our recently proposed novel biomarkers identified through chromogenic AP-RISH and z-proportion analysis [3]. Previously, we provided a detailed comparison of our data on biomarkers to work by Minogue et al. [42,43] and van den Akker et al. [44], as seen in Table 2 of Li et al. [3]. We hypothesized that RNA extraction from pooled cells as source for microarray or qRT-PCR transcriptome profiling could explain some of the noted differences especially for biomarkers identified through microarray technology [85]. An observation from an earlier study suggested Krt8, Krt18 and Krt19 as uniquely expressed in embryonic and fetal notochord cells in the human spine [86]. We identified *Krt8*, *Krt18* and *Krt19* positive cells in both outer AF and NP tissue of mature bovine IVDs by AP-RISH, yet only *Krt18* and *Krt19* were expressed by a significantly larger proportion of cells in the NP [3]. Other markers like *OVOS2*, *GDF10, GPC3*, *HBB* or *NCAM1,* which were identified as human NP-specific over articular cartilage (AC) were not part of our initial panel of 50 genes and not investigated in our studies [3,13,15,42,43,61,62,87,88]. Classically known markers of the chondrogenic lineage like *Col2a1*, *Acan* and *Sox9* have often been used to identify mature NP cells [13,89], yet taking cellular heterogeneity into account, *Sox9* was not detected in a significantly higher proportion of cells in the NP over the AF in our previous study [3]. Recently, a different set of biomarkers was proposed based on a meta-analysis of the membranome, comparing transcripts of retrovirally immortalized, clonal cell lines derived through enzymatic tissue digestion with datasets of primary human AF and NP cells acquired in an earlier study [81]. The study proposed *CLDN11, TMEFF2, CA12, ANAX2, CD44, EFNA1, NETO2* and *SLC2A1* as NP-specific cell surface markers to assess cellular heterogeneity of NP cells in vivo and suggested *CHIC1, COLEC12, LPAR1, LRP4, LRP5* and *FZD2* as markers to define cellular heterogeneity in AF cells. The immortalized NP cell lines were previously employed by this group to identify human NP subpopulations through qRT-PCR based expression profiling, pointing to *FOXF1* and *CA12* as useful markers with significant difference between clonal subtypes [90]. The approach to focus on the membranome is helpful as cell sorting based on cell surface proteins is often used to isolate different cell types [91], however, effects on gene expression through random retroviral insertion as well as comparing expression profiles obtained through different array platforms can be challenging [85]. A different microarray-based transcriptome analysis of human embryonic and fetal notochord cells recently aimed to identify regulators of IVD development [84]. The identified notochord specific markers *CD24, STMN2, RTN1, PRPH, CXC12, IGF1, ISL1, CLDN1, THBS2* and *MAP1B* were mostly down regulated in adult NP cells, with the exception of the latter. Using cell pooling for RNA extraction and genome wide microarray analysis another recent study also focused on the molecular discrimination between human AF and NP tissue suggesting six novel NP markers: *CDKN2B, ARAP2, ERFE, DSC3, DEFB1, SPTLC3* and five novel AF markers: *OLFML2A, ANKRD29, EMCN, ADGRL4* and *LDB2* with a significant difference in expression levels between the two tissue types [14]. While we confirmed several existing biomarkers established by others through methods employing cell pooling, the minimal overlap with biomarkers of recent studies is not surprising, as the methods, tissue sources and objectives varied between study designs and none of these more recently proposed biomarkers other than *Ca12* overlapped with our initial panel of 50 investigated genes [3], which led to the further investigation of the nine biomarkers *Lam1*, *Thy1*, *Gli1*, *Gli3*, *Noto*, *Ptprc*, *Scx*, *Sox2* and *Zscan10* presented here. While we cannot draw any further conclusions at this point, it would be interesting to investigate the novel markers proposed in the studies above in the context of cellular heterogeneity going forward.

Lastly, the combination of FL-RISH and the high resolution of confocal imaging allows for a glimpse at sub-cellular transcript location. It is known for some transcripts, like non-coding RNAs, that transcript location could point to regulatory functions [92]. In case of protein-coding mRNAs, an association between cytoplasmic location and function is also considered and found conserved between bacteria, yeast and higher eukaryotic cells [93,94,95]. Less information is available regarding a role of nuclear locations of primary RNA transcripts during processing prior to the export into the cytoplasm for translation. However sub-nuclear hotspots like the nucleoi for ribosomal RNA synthesis and assembly [96] or nuclear speckles, affiliated with the pre-mRNA splicing machinery [97] have been described. Interesting transcript accumulations as seen for the transcription factors *Sox2* (NP), *Zscan10* (AF, NP) or *Gli3* (AF, NP) could point to the proximity of such functional nuclear domains and possibly indicate active transcription and processing of transcripts. The novel markers *Lam1*, *Thy1*, *Gli1*, *Gli3*, *Noto*, *Ptprc*, *Scx*, *Sox2* and *Zscan10* suggested here in our study, should be considered as addition to a growing repertoire of biomarker panels for future characterization of outer AF and NP cells along with morphological and cell behavior data, like cell movement, velocity and clustering ability [2].

## 5. Conclusions

Future regenerative cell-based therapies are of great potential if a safe outcome is predictable. Whole genome transcriptome analysis is a powerful screening tool to identify novel biomarkers, however phenotyping and genotyping of heterogeneous cell populations with single-cell assessment is an important quality control measure in achieving this predictability. Animal research models like the bovine IVD are invaluable tools to better characterize resident cells within a healthy IVD environment. FL-RISH in combination with confocal microscopy and statistical data analysis can provide both, transcript analysis through population averaging and on a single-cell level. Both were used here to confirm our previously proposed biomarkers *Lam1*, *Thy1*, *Gli1*, *Gli3*, *Noto*, *Ptprc*, *Scx*, *Sox2* and *Zscan10*. We believe that a combination of the many biomarkers that have been established so far and those that will be determined in the future will be helpful to clarify the molecular identity of cells in the mature IVD, however, data re-assessment from a cell-by-cell perspective taking heterogeneity into account might become necessary. 

## Figures and Tables

**Table 1:** Comparison of NP/AF ratio data for nine proposed biomarkers in cells of the outer annulus fibrosus (AF) and nucleus pulposus (NP) of a mature bovine IVD generated through z-proportion test and population averaging comparing data from Li et al [3] using RNA in situ hybridization (AP-RISH) (purple) and this work using Fluorescent RNA in situ hybridization (FL-RISH) (green). Laminin1 (*Lam1*); Thymocyte differentiation antigen 1 (*Thy1*); Glioma-associated oncogene 1 (*Gli1*); Glioma-associated oncogene 3 (*Gli3*); Notochord (*Noto*); Tyrosine phosphate receptor type C (*Ptprc*); Scleraxis (*Scx*); Sex determining region Y-box 2 (*Sox2*); Zinc finger and SCAN (*SRE-ZBP, CTfin51, AW-1* and Number 18 cDNA) domain containing transcription factor 10 (*Zscan10*).

Biomarker	AP-RISH and z-Proportion Test	FL-RISH and z-Proportion Test	FL-RISH and Population Averaging
	NP/AF Ratio	NP/AF Ratio	FL in NP/FL in AF Ratio
*Lam1*	0.3	0.8	0.6
*Thy1*	0.2	0.7	0.5
*Gli1*	2.2	2.0	3.8
*Gli3*	1.5	2.0	3.3
*Noto*	4.3	2.4	2.7
*Ptprc*	2.3	2.2	2.6
*Scx*	2.2	3.4	10.8
*Sox2*	3.2	2.2	2.1
*Zscan10*	1.6	2.0	2.5

## Supplementary Materials

The following are available online at https://www.mdpi.com/2306-7381/6/2/42/s1, Figure S1: Kernel density estimates by intervertebral disc (IVD) for nine proposed biomarkers in (A) cells of the outer annulus fibrosus (AF) and (B) nucleus pulposus (NP) derived from three sections of a mature bovine IVD. Laminin1 (*Lam1*); Thymocyte differentiation antigen 1 (*Thy1*); Glioma-associated oncogene 1 (*Gli1*); Glioma-associated oncogene 3 (*Gli3*); Notochord (*Noto*); Tyrosine phosphate receptor type C (*Ptprc*); Scleraxis (*Scx*); Sex determining region Y-box 2 (*Sox2*); Zinc finger and SCAN domain containing (*Zscan10*). Table S1: Data for the population average of transcript expression for nine proposed biomarkers in cells of the outer annulus fibrosus (AF) and nucleus pulposus (NP) of a mature bovine IVD. Laminin1 (*Lam1*); Thymocyte differentiation antigen 1 (*Thy1*); Glioma-associated oncogene 1 (*Gli1*); Glioma-associated oncogene 3 (*Gli3*); Notochord (*Noto*); Tyrosine phosphate receptor type C (*Ptprc*); Scleraxis (*Scx*); Sex determining region Y-box 2 (*Sox2*); Zinc finger and SCAN domain containing (*Zscan10*). Table S2: Descriptive statistics and normality tests for total fluorescence and log-transformed total fluorescence for nine proposed biomarkers in cells of the outer AF and NP of a mature bovine IVD. Shapiro–Wilk tests reveal that log-transformed data is more normally distributed, while k-means clustering is implemented to fit the other data to a Gaussian mixture model. Cluster descriptive statistics are reported when data is not normally distributed. Laminin1 (*Lam1*); Thymocyte differentiation antigen 1 (*Thy1*); Glioma-associated oncogene 1 (*Gli1*); Glioma-associated oncogene 3 (*Gli3*); Notochord (*Noto*); Tyrosine phosphate receptor type C (*Ptprc*); Scleraxis (*Scx*); Sex determining region Y-box 2 (*Sox2*); Zinc finger and SCAN domain containing (*Zscan10*). Table S3: Data for kernel density estimates by IVD for nine proposed biomarkers. Laminin1 (*Lam1*); Thymocyte differentiation antigen 1 (*Thy1*); Glioma-associated oncogene 1 (*Gli1*); Glioma-associated oncogene 3 (*Gli3*); Notochord (*Noto*); Tyrosine phosphate receptor type C (*Ptprc*); Scleraxis (*Scx*); Sex determining region Y-box 2 (*Sox2*); Zinc finger and SCAN domain containing (*Zscan10*).

## References

[1] Humzah M.D., Soames R.W. (1988). Human intervertebral disc: structure and function. Anat Rec..

[2] Kraus P., Li K., Sipes D., Varden L., Yerden R., Henderson A., Sur S., Lufkin T., Santra T.S., Tseng F.-G. (2019). Single-Cell Phenotyping of Complex Heterogeneous Tissue. Handbook of Single Cell Technologies.

[3] Li K., Kapper D., Youngs B., Kocsis V., Mondal S., Kraus P., Lufkin T. (2019). Potential biomarkers of the mature intervertebral disc identified at the single cell level. J. Anat..

[4] Stergar J., Gradisnik L., Velnar T., Maver U. (2019). Intervertebral disc tissue engineering: A brief review. Bosn. J. Basic. Med. Sci..

[5] Smith L.J., Silverman L., Sakai D., Le Maitre C.L., Mauck R.L., Malhotra N.R., Lotz J.C., Buckley C.T. (2018). Advancing cell therapies for intervertebral disc regeneration from the lab to the clinic: Recommendations of the ORS spine section. JOR Spine.

[6] McCann M.R., Bacher C.A., Seguin C.A. (2011). Exploiting notochord cells for stem cell-based regeneration of the intervertebral disc. J. Cell Commun. Signal.

[7] Eyre D.R., Muir H. (1976). Types I and II collagens in intervertebral disc. Interchanging radial distributions in annulus fibrosus. Biochem. J..

[8] Bron J.L., Helder M.N., Meisel H.J., Van Royen B.J., Smit T.H. (2009). Repair, regenerative and supportive therapies of the annulus fibrosus: achievements and challenges. Eur. Spine J..

[9] Sivakamasundari V., Lufkin T. (2013). Stemming the Degeneration: IVD Stem Cells and Stem Cell Regenerative Therapy for Degenerative Disc Disease. Adv. Stem. Cells.

[10] Pennicooke B., Moriguchi Y., Hussain I., Bonssar L., Hartl R. (2016). Biological Treatment Approaches for Degenerative Disc Disease: A Review of Clinical Trials and Future Directions. Cureus.

[11] Oehme D., Goldschlager T., Ghosh P., Rosenfeld J.V., Jenkin G. (2015). Cell-Based Therapies Used to Treat Lumbar Degenerative Disc Disease: A Systematic Review of Animal Studies and Human Clinical Trials. Stem Cells Int..

[12] Brown C. (2012). Stem cell tourism poses risks. CMAJ.

[13] Rodrigues-Pinto R., Richardson S.M., Hoyland J.A. (2013). Identification of novel nucleus pulposus markers: Interspecies variations and implications for cell-based therapiesfor intervertebral disc degeneration. Bone Joint Res..

[14] Schubert A.K., Smink J.J., Arp M., Ringe J., Hegewald A.A., Sittinger M. (2018). Quality Assessment of Surgical Disc Samples Discriminates Human Annulus Fibrosus and Nucleus Pulposus on Tissue and Molecular Level. Int. J. Mol. Sci..

[15] Rutges J., Creemers L.B., Dhert W., Milz S., Sakai D., Mochida J., Alini M., Grad S. (2010). Variations in gene and protein expression in human nucleus pulposus in comparison with annulus fibrosus and cartilage cells: potential associations with aging and degeneration. Osteoarthritis Cartilage.

[16] Kraus P., Sivakamasundari V., Xing X., Lufkin T. (2014). Generating mouse lines for lineage tracing and knockout studies. Methods Mol. Biol..

[17] Choi K.S., Harfe B.D. (2011). Hedgehog signaling is required for formation of the notochord sheath and patterning of nuclei pulposi within the intervertebral discs. PNAS.

[18] Choi K.S., Lee C., Harfe B.D. (2012). Sonic hedgehog in the notochord is sufficient for patterning of the intervertebral discs. Mech. Dev..

[19] Smits P., Lefebvre V. (2003). Sox5 and Sox6 are required for notochord extracellular matrix sheath formation, notochord cell survival and development of the nucleus pulposus of intervertebral discs. Development.

[20] Kraus P., Yerden R., Kocsis V., Lufkin T. (2017). RNA in situ hybridization characterization of non-enzymatic derived bovine intervertebral disc cell lineages suggests progenitor cell potential. Acta Histochem..

[21] Oshima H., Ishihara H., Urban J.P., Tsuji H. (1993). The use of coccygeal discs to study intervertebral disc metabolism. J. Orthop. Res..

[22] Demers C.N., Antoniou J., Mwale F. (2004). Value and limitations of using the bovine tail as a model for the human lumbar spine. Spine (Phila Pa 1976).

[23] Beckstein J.C., Sen S., Schaer T.P., Vresilovic E.J., Elliott D.M. (2008). Comparison of animal discs used in disc research to human lumbar disc: axial compression mechanics and glycosaminoglycan content. Spine (Phila Pa 1976).

[24] Michalek A.J. (2019). A growth-based model for the prediction of fiber angle distribution in the intervertebral disc annulus fibrosus. Biomech. Model Mechanobiol..

[25] Ekblom M., Falk M., Salmivirta K., Durbeej M., Ekblom P. (1998). Laminin isoforms and epithelial development. Ann. N. Y. Acad. Sci..

[26] Ahn S., Joyner A.L. (2005). In vivo analysis of quiescent adult neural stem cells responding to Sonic hedgehog. Nature.

[27] Buttitta L., Mo R., Hui C.C., Fan C.M. (2003). Interplays of Gli2 and Gli3 and their requirement in mediating Shh-dependent sclerotome induction. Development.

[28] Shin S.H., Kogerman P., Lindstrom E., Toftgard R., Biesecker L.G. (1999). GLI3 mutations in human disorders mimic Drosophila cubitus interruptus protein functions and localization. PNAS.

[29] Abdelkhalek H.B., Beckers A., Schuster-Gossler K., Pavlova M.N., Burkhardt H., Lickert H., Rossant J., Reinhardt R., Schalkwyk L.C., Muller I. (2004). The mouse homeobox gene Not is required for caudal notochord development and affected by the truncate mutation. Genes Dev..

[30] McCann M.R., Tamplin O.J., Rossant J., Seguin C.A. (2012). Tracing notochord-derived cells using a Noto-cre mouse: Implications for intervertebral disc development. Dis. Model Mech..

[31] Cserjesi P., Brown D., Ligon K.L., Lyons G.E., Copeland N.G., Gilbert D.J., Jenkins N.A., Olson E.N. (1995). Scleraxis: A basic helix-loop-helix protein that prefigures skeletal formation during mouse embryogenesis. Development.

[32] Shukunami C., Takimoto A., Oro M., Hiraki Y. (2006). Scleraxis positively regulates the expression of tenomodulin, a differentiation marker of tenocytes. Dev. Biol..

[33] Niwa H., Ogawa K., Shimosato D., Adachi K. (2009). A parallel circuit of LIF signalling pathways maintains pluripotency of mouse ES cells. Nature.

[34] Takahashi K., Yamanaka S. (2006). Induction of pluripotent stem cells from mouse embryonic and adult fibroblast cultures by defined factors. Cell.

[35] Kraus P., Sivakamasundari V., Yu H.B., Xing X., Lim S.L., Adler T., Pimentel J.A., Becker L., Bohla A., Garrett L. (2014). Pleiotropic functions for transcription factor zscan10. PLoS ONE.

[36] Dominici M., Le Blanc K., Mueller I., Slaper-Cortenbach I., Marini F., Krause D., Deans R., Keating A., Prockop D., Horwitz E. (2006). Minimal criteria for defining multipotent mesenchymal stromal cells. The International Society for Cellular Therapy position statement. Cytotherapy.

[37] Lyu F.J., Cheung K.M., Zheng Z., Wang H., Sakai D., Leung V.Y. (2019). IVD progenitor cells: a new horizon for understanding disc homeostasis and repair. Nat. Rev. Rheumatol..

[38] Wang W., Lo P., Frasch M., Lufkin T. (2000). Hmx: an evolutionary conserved homeobox gene family expressed in the developing nervous system in mice and Drosophila. Mech. Dev..

[39] Lohnes D., Dierich A., Ghyselinck N., Kastner P., Lampron C., LeMeur M., Lufkin T., Mendelsohn C., Nakshatri H., Chambon P. (1992). Retinoid receptors and binding proteins. J. Cell Sci. Suppl..

[40] Kraus P., Lufkin T. (1999). Mammalian Dlx homeobox gene control of craniofacial and inner ear morphogenesis. J. Cell Biochem..

[41] Chen X., Li X., Wang W., Lufkin T. (1996). Dlx5 and Dlx6: an evolutionary conserved pair of murine homeobox genes expressed in the embryonic skeleton. Ann. N. Y. Acad. Sci..

[42] Minogue B.M., Richardson S.M., Zeef L.A., Freemont A.J., Hoyland J.A. (2010). Transcriptional profiling of bovine intervertebral disc cells: implications for identification of normal and degenerate human intervertebral disc cell phenotypes. Arthritis Res. Ther..

[43] Minogue B.M., Richardson S.M., Zeef L.A., Freemont A.J., Hoyland J.A. (2010). Characterization of the human nucleus pulposus cell phenotype and evaluation of novel marker gene expression to define adult stem cell differentiation. Arthritis Rheum..

[44] Van den Akker G.G.H., Koenders M.I., van de Loo F.A.J., van Lent P., Blaney Davidson E., van der Kraan P.M. (2017). Transcriptional profiling distinguishes inner and outer annulus fibrosus from nucleus pulposus in the bovine intervertebral disc. Eur. Spine J..

[45] Chatterjee S., Kraus P., Sivakamasundari V., Xing X., Yap S.P., Jie S., Lufkin T. (2013). A conditional mouse line for lineage tracing of Sox9 loss-of-function cells using enhanced green fluorescent protein. Biotechnol. Lett..

[46] Kraus P., Sivakamasundari V., Olsen V., Villeneuve V., Hinds A., Lufkin T. (2019). Klhl14 antisense RNA is a target of key skeletogenic transcription factors in the developing intervertebral disc. Spine (Phila Pa 1976).

[47] Chatterjee S., Sivakamasundari V., Yap S.P., Kraus P., Kumar V., Xing X., Lim S.L., Sng J., Prabhakar S., Lufkin T. (2014). In vivo genome-wide analysis of multiple tissues identifies gene regulatory networks, novel functions and downstream regulatory genes for Bapx1 and its co-regulation with Sox9 in the mammalian vertebral column. BMC Genomics.

[48] Lee W.J., Chatterjee S., Yap S.P., Lim S.L., Xing X., Kraus P., Sun W., Hu X., Sivakamasundari V., Chan H.Y. (2017). An Integrative Developmental Genomics and Systems Biology Approach to Identify an In Vivo Sox Trio-Mediated Gene Regulatory Network in Murine Embryos. Biomed. Res. Int..

[49] Sivakamasundari V., Kraus P., Jie S., Lufkin T. (2013). Pax1(EGFP): New wildtype and mutant EGFP mouse lines for molecular and fate mapping studies. Genesis.

[50] Sivakamasundari V., Kraus P., Sun W., Hu X., Lim S.L., Prabhakar S., Lufkin T. (2017). A developmental transcriptomic analysis of Pax1 and Pax9 in embryonic intervertebral disc development. Biol. Open.

[51] Murray C.J., Atkinson C., Bhalla K., Birbeck G., Burstein R., Chou D., Dellavalle R., Danaei G., Ezzati M., Fahimi A. (2013). The state of US health, 1990-2010: burden of diseases, injuries, and risk factors. JAMA.

[52] Daly C., Ghosh P., Jenkin G., Oehme D., Goldschlager T. (2016). A Review of Animal Models of Intervertebral Disc Degeneration: Pathophysiology, Regeneration, and Translation to the Clinic. Biomed. Res. Int..

[53] Kraus P., Lufkin T. (2017). Implications for a Stem Cell Regenerative Medicine Based Approach to Human Intervertebral Disk Degeneration. Front. Cell Dev. Biol..

[54] Lyons G., Eisenstein S.M., Sweet M.B. (1981). Biochemical changes in intervertebral disc degeneration. Biochim. Biophys. Acta.

[55] Antoniou J., Steffen T., Nelson F., Winterbottom N., Hollander A.P., Poole R.A., Aebi M., Alini M. (1996). The human lumbar intervertebral disc: evidence for changes in the biosynthesis and denaturation of the extracellular matrix with growth, maturation, ageing, and degeneration. J. Clin. Invest..

[56] Urban J.P., Roberts S. (2003). Degeneration of the intervertebral disc. Arthritis Res. Ther..

[57] Sakai D., Andersson G.B. (2015). Stem cell therapy for intervertebral disc regeneration: obstacles and solutions. Nat. Rev. Rheumatol..

[58] Lama P., Le Maitre C.L., Harding I.J., Dolan P., Adams M.A. (2018). Nerves and blood vessels in degenerated intervertebral discs are confined to physically disrupted tissue. J. Anat..

[59] Zhang C., Wang B., Zhao X., Li X., Lou Z., Chen X., Zhang F. (2018). Iron deficiency accelerates intervertebral disc degeneration through affecting the stability of DNA polymerase epsilon complex. Am. J. Transl. Res..

[60] Haidar R., Musallam K.M., Taher A.T. (2011). Bone disease and skeletal complications in patients with beta thalassemia major. Bone.

[61] Risbud M.V., Schoepflin Z.R., Mwale F., Kandel R.A., Grad S., Iatridis J.C., Sakai D., Hoyland J.A. (2015). Defining the phenotype of young healthy nucleus pulposus cells: Recommendations of the Spine Research Interest Group at the 2014 annual ORS meeting. J. Orthop. Res..

[62] Risbud M.V., Shapiro I.M. (2011). Notochordal cells in the adult intervertebral disc: new perspective on an old question. Crit. Rev. Eukaryot. Gene Expr..

[63] Bushell G.R., Ghosh P., Taylor T.F., Akeson W.H. (1977). Proteoglycan chemistry of the intervertebral disks. Clin. Orthop. Relat. Res..

[64] Sng J., Lufkin T. (2012). Emerging stem cell therapies: treatment, safety, and biology. Stem Cells Int..

[65] Urban J.P., Holm S., Maroudas A., Nachemson A. (1977). Nutrition of the intervertebral disk. An in vivo study of solute transport. Clin. Orthop. Relat. Res..

[66] Wuertz K., Godburn K., Neidlinger-Wilke C., Urban J., Iatridis J.C. (2008). Behavior of mesenchymal stem cells in the chemical microenvironment of the intervertebral disc. Spine (Phila Pa 1976).

[67] Liang C.Z., Li H., Tao Y.Q., Zhou X.P., Yang Z.R., Li F.C., Chen Q.X. (2012). The relationship between low pH in intervertebral discs and low back pain: a systematic review. Arch. Med. Sci..

[68] Choi H., Johnson Z.I., Risbud M.V. (2015). Understanding nucleus pulposus cell phenotype: a prerequisite for stem cell based therapies to treat intervertebral disc degeneration. Curr. Stem Cell Res. Ther..

[69] Errington R.J., Puustjarvi K., White I.R., Roberts S., Urban J.P. (1998). Characterisation of cytoplasm-filled processes in cells of the intervertebral disc. J. Anat..

[70] Huang Y.C., Urban J.P., Luk K.D. (2014). Intervertebral disc regeneration: do nutrients lead the way?. Nat. Rev. Rheumatol..

[71] Bedore J., Leask A., Seguin C.A. (2014). Targeting the extracellular matrix: matricellular proteins regulate cell-extracellular matrix communication within distinct niches of the intervertebral disc. Matrix. Biol..

[72] Walmsley R. (1953). The development and growth of the intervertebral disc. Edinb. Med. J..

[73] Trout J.J., Buckwalter J.A., Moore K.C. (1982). Ultrastructure of the human intervertebral disc: II. Cells of the nucleus pulposus. Anat. Rec..

[74] Trout J.J., Buckwalter J.A., Moore K.C., Landas S.K. (1982). Ultrastructure of the human intervertebral disc. I. Changes in notochordal cells with age. Tissue Cell.

[75] Chen J., Yan W., Setton L.A. (2006). Molecular phenotypes of notochordal cells purified from immature nucleus pulposus. Eur. Spine J..

[76] Tanaka M., Sakai D., Hiyama A., Arai F., Nakajima D., Yokoyama K., Mochida J. (2012). Evidence of Nonnotochordal Origin in Chondrocyte-like Cells of the Nucleus Pulposus Appearing in Early Stage Disk Degeneration in the Mouse Model. Global Spine J..

[77] Kraus P., Lufkin T. (2016). Bovine annulus fibrosus cell lines isolated from intervertebral discs. Genom. Data.

[78] Kraus P., Kocsis V., Williams C., Youngs B., Lufkin T. (2015). Plate in situ hybridization (PISH) as a time and cost effective RNA expression assay to study phenotypic heterogeneity in a population of cultured murine cells at single cell resolution. Biotechnol. Lett..

[79] Kraus P., Yerden R., Sipes D., Sur S., Lufkin T. (2018). A quantitative and qualitative RNA expression profiling assay for cell culture with single cell resolution. Cytotechnology.

[80] Thorpe A.A., Binch A.L., Creemers L.B., Sammon C., Le Maitre C.L. (2016). Nucleus pulposus phenotypic markers to determine stem cell differentiation: fact or fiction?. Oncotarget.

[81] Akker G., Eijssen L.M.T., Richardson S.M., Rhijn L.W.V., Hoyland J.A., Welting T.J.M., Voncken J.W. (2018). A Membranome-Centered Approach Defines Novel Biomarkers for Cellular Subtypes in the Intervertebral Disc. Cartilage.

[82] Scholzen T., Gerdes J. (2000). The Ki-67 protein: from the known and the unknown. J. Cell Physiol..

[83] Gerdes J., Schwab U., Lemke H., Stein H. (1983). Production of a mouse monoclonal antibody reactive with a human nuclear antigen associated with cell proliferation. Int. J. Cancer.

[84] Rodrigues-Pinto R., Ward L., Humphreys M., Zeef L.A.H., Berry A., Hanley K.P., Hanley N., Richardson S.M., Hoyland J.A. (2018). Human notochordal cell transcriptome unveils potential regulators of cell function in the developing intervertebral disc. Sci. Rep..

[85] Kraus P., Xing X., Lim S.L., Fun M.E., Sivakamasundari V., Yap S.P., Lee H., Karuturi R.K., Lufkin T. (2012). Mouse strain specific gene expression differences for illumina microarray expression profiling in embryos. BMC Res. Notes.

[86] Rodrigues-Pinto R., Berry A., Piper-Hanley K., Hanley N., Richardson S.M., Hoyland J.A. (2016). Spatiotemporal analysis of putative notochordal cell markers reveals CD24 and keratins 8, 18, and 19 as notochord-specific markers during early human intervertebral disc development. J. Orthop. Res..

[87] Lv F., Leung V.Y., Huang S., Huang Y., Sun Y., Cheung K.M. (2014). In search of nucleus pulposus-specific molecular markers. Rheumatology (Oxford).

[88] Power K.A., Grad S., Rutges J.P., Creemers L.B., van Rijen M.H., O’Gaora P., Wall J.G., Alini M., Pandit A., Gallagher W.M. (2011). Identification of cell surface-specific markers to target human nucleus pulposus cells: expression of carbonic anhydrase XII varies with age and degeneration. Arthritis Rheum..

[89] Sive J.I., Baird P., Jeziorsk M., Watkins A., Hoyland J.A., Freemont A.J. (2002). Expression of chondrocyte markers by cells of normal and degenerate intervertebral discs. Mol. Pathol..

[90] Van den Akker G.G., Surtel D.A., Cremers A., Rodrigues-Pinto R., Richardson S.M., Hoyland J.A., van Rhijn L.W., Welting T.J., Voncken J.W. (2014). Novel immortal human cell lines reveal subpopulations in the nucleus pulposus. Arthritis Res. Ther..

[91] Risbud M.V., Guttapalli A., Tsai T.T., Lee J.Y., Danielson K.G., Vaccaro A.R., Albert T.J., Gazit Z., Gazit D., Shapiro I.M. (2007). Evidence for skeletal progenitor cells in the degenerate human intervertebral disc. Spine (Phila Pa 1976).

[92] Salviano-Silva A., Lobo-Alves S.C., Almeida R.C., Malheiros D., Petzl-Erler M.L. (2018). Besides Pathology: Long Non-Coding RNA in Cell and Tissue Homeostasis. Noncoding RNA.

[93] Buxbaum A.R., Haimovich G., Singer R.H. (2015). In the right place at the right time: visualizing and understanding mRNA localization. Nat. Rev. Mol. Cell. Biol..

[94] Eliscovich C., Singer R.H. (2017). RNP transport in cell biology: the long and winding road. Curr. Opin. Cell Biol..

[95] Mofatteh M., Bullock S.L. (2017). SnapShot: Subcellular mRNA Localization. Cell.

[96] Sirri V., Urcuqui-Inchima S., Roussel P., Hernandez-Verdun D. (2008). Nucleolus: the fascinating nuclear body. Histochem. Cell Biol..

[97] Spector D.L., Lamond A.I. (2011). Nuclear speckles. Cold Spring Harb. Perspect. Biol..

