# Supplementary material for: Quantitative Single-Cell Transcript Assessment of Biomarkers Supports Cellular Heterogeneity in the Bovine IVD
Source: Vet Sci. 2019 May 12;6(2):42. doi: 10.3390/vetsci6020042 (PMC6631975; doi:10.3390/vetsci6020042)
Supplement: Supplementary file 1 [file vetsci-06-00042-s001.zip › Supplementary Material/Supplementary Materials Legends.docx]

**Supplementary Materials:** The following are available online at [www.mdpi.com/xxx/s1](http://www.mdpi.com/xxx/s1),

Figure S1. Kernel density estimates by IVD for nine proposed biomarkers in (A) cells of the outer annulus fibrosus (AF) and (B) nucleus pulposus (NP) derived from three sections of a mature bovine IVD. *Laminin1* (*Lam1*); *Thymocyte differentiation antigen 1* (*Thy1*); *Glioma-associated oncogene 1* (*Gli1*); *Glioma-associated oncogene 3* (*Gli3*); *Notochord* (*Noto*); *Tyrosine phosphate receptor type C* (*Ptprc*); *Scleraxis* (*Sxc*); *Sex determining region Y-box 2* (*Sox2*); *Zinc finger and SCAN domain containing* (*Zscan10*).

Table S1. Data for the population average of transcript expression for nine proposed biomarkers in cells of the outer annulus fibrosus (AF) and nucleus pulposus (NP) of a mature bovine IVD. *Laminin1* (*Lam1*); *Thymocyte differentiation antigen 1* (*Thy1*); *Glioma-associated oncogene 1* (*Gli1*); *Glioma-associated oncogene 3* (*Gli3*); *Notochord* (*Noto*); *Tyrosine phosphate receptor type C* (*Ptprc*); *Scleraxis* (*Sxc*); *Sex determining region Y-box 2* (*Sox2*); *Zinc finger and SCAN domain containing* (*Zscan10*).

Table S2. Descriptive statistics and normality tests for total fluorescence and log-transformed total fluorescence for nine proposed biomarkers in cells of the outer AF and NP of a mature bovine IVD. Shapiro-Wilk tests reveal that log-transformed data is more normally distributed, while k-means clustering is implemented to fit the other data to a Gaussian mixture model. Cluster descriptive statistics are reported when data is not normally distributed. *Laminin1* (*Lam1*); *Thymocyte differentiation antigen 1* (*Thy1*); *Glioma-associated oncogene 1* (*Gli1*); *Glioma-associated oncogene 3* (*Gli3*); *Notochord* (*Noto*); *Tyrosine phosphate receptor type C* (*Ptprc*); *Scleraxis* (*Sxc*); *Sex determining region Y-box 2* (*Sox2*); *Zinc finger and SCAN domain containing* (*Zscan10*).

Table S2. Data for Kernel density estimates by IVD for nine proposed biomarkers. *Laminin1* (*Lam1*); *Thymocyte differentiation antigen 1* (*Thy1*); *Glioma-associated oncogene 1* (*Gli1*); *Glioma-associated oncogene 3* (*Gli3*); *Notochord* (*Noto*); *Tyrosine phosphate receptor type C* (*Ptprc*); *Scleraxis* (*Sxc*); *Sex determining region Y-box 2* (*Sox2*); *Zinc finger and SCAN domain containing* (*Zscan10*).
