# Supplementary figures and images for: Quantitative Single-Cell Transcript Assessment of Biomarkers Supports Cellular Heterogeneity in the Bovine IVD
Source: Vet Sci. 2019 May 12;6(2):42. doi: 10.3390/vetsci6020042 (PMC6631975; doi:10.3390/vetsci6020042)

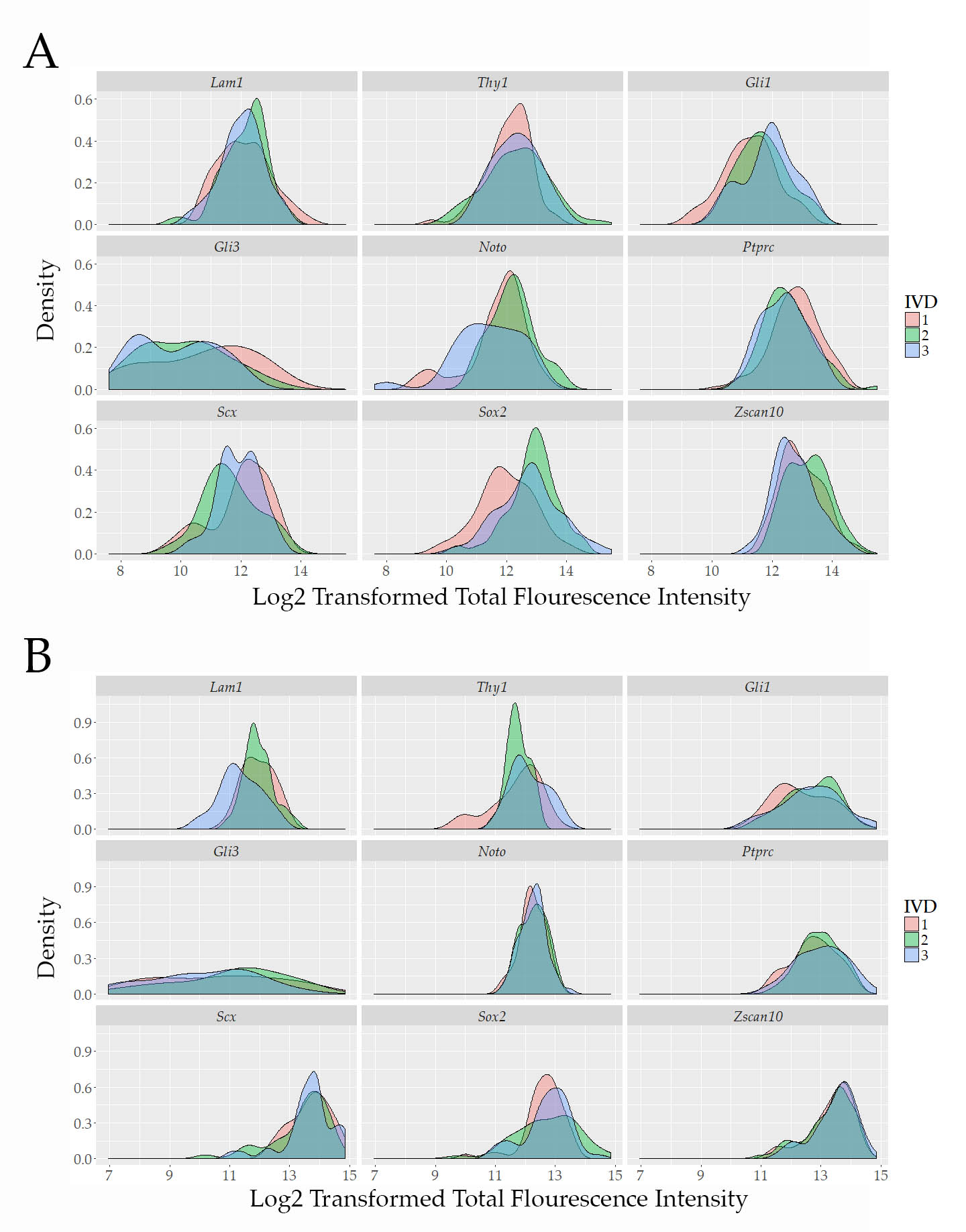

Supplement: Supplementary file 1 [file vetsci-06-00042-s001.zip › Supplementary Material/Figure S1.jpg]
